# Effect of High-Hydrostatic-Pressure Treatment on the Physicochemical Properties of Kafirin

**DOI:** 10.3390/foods12224077

**Published:** 2023-11-09

**Authors:** Yajing Yang, Qiongling Chen, Qingshan Liu, Xiaowen Wang, Wenbin Bai, Zhenjia Chen

**Affiliations:** 1 College of Food Science and Engineering, Shanxi Agricultural University, No. 1, Mingxian South Road, Taigu Direct, Jinzhong 030801, China; y13233483103@163.com (Y.Y.); cql_ttxs@163.com (Q.C.); wwxw11@163.com (X.W.); 2 The Sorghum Research Institute, Shanxi Agricultural University, No. 238, Yunhua West Road, Yuci Direct, Jinzhong 030600, China; 673673@126.com (Q.L.); baiwenbin1983@126.com (W.B.)

**Keywords:** disulfide bonds, high hydrostatic pressure, kafirin, SDS-PAGE

## Abstract

The kafirin derived from Jin Nuo 3 sorghum underwent a high-hydrostatic-pressure (HHP) treatment of 100, 300, and 600 MPa for 10 min to investigate alterations in its physicochemical attributes. The findings exhibited a reduction in protein solubility, declining from 83% to 62%, consequent to the application of the HHP treatment. However, this treatment did not lead to subunit-specific aggregation. The absorption intensity of UV light diminished, and the peak fluorescence absorption wavelength exhibited a shift from 342 nm to 344 nm, indicating an increased polarity within the amino acid microenvironment. In an aqueous solution, the specific surface area expanded from 294.2 m^2^/kg to 304.5 m^2^/kg, while the average particle-size value in a 70% ethanol solution rose to 26.3 nm. Conversely, the zeta-potential value decreased from 3.4 mV to 1.3 mV, suggesting a propensity for aggregation in ethanol solutions. A notable rise in the intermolecular β-sheet content to 21.06% was observed, along with a shift in the peak denaturation temperature from 76.33 °C to 86.33 °C. Additionally, the content of disulfide bonds increased to 14.5 μmol/g. Collectively, the application of the HHP treatment not only enhanced the thermal stability but also induced a more ordered secondary structure within the kafirin.

## 1. Introduction

Sorghum (*Sorghum bicolor* (L.) Moench), in the family Gramineae, holds a significant position among the world’s five principal cereal crops, exerting a pivotal influence on the global food industry [1]. Sorghum occupies a central role because of its versatility. It is not only the main grain crop in arid and semiarid regions of the world, but it also plays a major role as fodder in richer countries. Moreover, sorghum contributes to the production of fuel, bioethanol, alcoholic beverages, and construction materials [2]. Within sorghum seeds, an array of essential nutrients abounds, encompassing fiber, protein, fats, carbohydrates, vitamins, phenols, and minerals. These nutrients confer a spectrum of biological effects, including antioxidant, antiobesity, antidiabetic, anticardiovascular, and anticancer properties [3]. Especially, sorghum boasts an approximate 11% protein content, dominated by kafirin, the principal storage protein. Kafirin constitutes a substantial portion, ranging from 70% to 90%, of the protein content [4]. Kafirin can be categorized into four distinct types: α-, β-, γ-, and δ-kafirin. Among these, α-kafirin claims the largest share, accounting for 70–80%, while β-kafirin contributes 7–8%, γ-kafirin comprises 9–12%, and δ-kafirin exhibits the lowest concentration at 1% [5].

Although kafirin is one of the most abundant of the sorghum proteins, its utilization has been limited by having low digestibility. The first reason is the grain organization of sorghum, where DURESSA et al. [6] determined the digestibility of the whole grain, endosperm, and the proteasomes themselves, respectively. The data demonstrated a gradual increase in sorghum protein digestibility as the structural complexity of the grain decreased, but the digestibility of the isolated proteasome fraction remained low. This indicates that the proteasome itself is highly resistant to digestion and is difficult to be digested and absorbed by the body. It is worth noting that the main reason for poor sorghum digestibility is the covalent cross-linking of kafirin through disulfide bonds. In the composition of kafirin, β- and γ-kafirin form a compact polymeric network, leaving the most easily digestible α-kafirin wrapped inside the protein and the last kafirin to be broken down. This prevents it from being fully digested, resulting in a low nutritional value [7]. The traditional way of cooking sorghum for porridge also reduces its digestibility. Studies have shown that disulfide bonds in the outer layer of sorghum bind to form a protein shell after cooking. Kafirin may form polymers through intermolecular disulfide bonding, resulting in a stronger structure in the inner layer at the periphery of the proteasome in response to heat. It is less likely to come into contact with enzymes and increase their resistance to digestion [8].

High hydrostatic pressure (HHP) stands as an emerging nonthermal technique, fostering the establishment or disruption of noncovalent bonds within food constituents. This action leads to the deactivation of enzymes, proteins, and starches, concurrently eradicating pathogenic bacteria and microorganisms present in the food matrix. Remarkably, this process upholds the food’s intrinsic attributes—color, flavor, nutritional value, and overall quality—while abstaining from the application of heat [9]. In recent times, in tandem with the widespread adoption of health-conscious dietary practices, there exists a heightened emphasis on the consumption of whole foods. Consequently, the preservation or augmentation of food’s nutritional worth has gained pronounced significance. Research reveals that the implementation of HHP technology exerts a positive influence on food’s antioxidant potential. This technology safeguards the integrity of antioxidant compounds in fruits, thus mitigating the degradation of vital nutrients. Moreover, it contributes to the regulation of blood glucose levels and the modulation of the glycemic index by promoting the accumulation of resistant starch within food items. Beyond these benefits, the extraction of nutrients from food is facilitated, a crucial aspect in the formulation of novel, health-enhancing food products [10].

The HHP technique involves subjecting proteins to pressure, which effectively disrupts relatively weak chemical bonds. This process not only influences protein–protein and protein–solvent interactions, but also induces alterations in the natural proteins’ conformation [11]. The resultant modification in protein structure extends to their properties, including solubility and propensity to form aggregates, which impacts their function within the realm of food processing [12]. In the study conducted by Zhang [13] et al., it was proven that treating with HHP led to an augmentation in the disulfide-bond content of millet gliadin. This alteration translated into a more organized protein structure and a conspicuously elevated level of thermal stability. Similarly, Kieffer [14] et al., while subjecting wheat gliadin to the HHP treatment, observed shifts in the secondary structure and a reduction in viscosity, implying significant changes in its physical characteristics.

Due to its strong hydrophobicity and low digestion, kafirin offers a lot of potential as edible and biodegradable coatings and films. With constant modification studies, researchers have achieved significant progress in film-forming agents to produce kafirin films with improved tensile strength and water repellency. Furthermore, it can be utilized as an encapsulant to provide antioxidant actives that will be released in specific areas to have particular effects [15]. Since HHP has a modifying influence on proteins, this technique was used on kafirin to overcome its limited digestibility, which has been limiting its use in food processing. To discover how this technique affected the physicochemical characteristics of kafirin, and to offer a reference for its implementation in the food-processing industry, HHP was applied to kafirin in this study.

## 2. Materials and Methods

### 2.1. Materials

Sorghum seeds of the Jin Nuo 3 variety were chosen from China for this study. Essential chemicals, including sodium metabisulfite, sodium hydroxide, urea, potassium bromide, disodium ethylenediaminetetraacetic acid, copper sulfate, potassium sulfate, sulfuric acid, and hydrochloric acid, were sourced from Sinopharm Chemical Reagents Co., Ltd. (Shanghai, China). For experimentation, low-molecular-weight protein markers (14.4–97.4 ku), β-sulfanylethanol, bromophenol blue, trimethylolaminomethane (Tris), sodium dodecyl sulfate (SDS), glycine, tetraethyl ethylenediamine, dithioerythritol (DTE), and ammonium persulfate, were procured from Beijing Solarbio Science & Technology Co., Ltd. (Beijing, China). The necessary 5,5-dithiobis (2-nitrobenzoic acid) (DTNB) was supplied by Aladdin Biochemical Technology Co., Ltd. (Shanghai, China). Petroleum ether and anhydrous ethanol were provided by Tianjin Beichen Founder Reagent Factory (Tianjin, China) to facilitate the experimental processes. Additional materials, including glacial acetic acid, acrylamide, and methylene bisacrylamide, were acquired from Sangon Biotech Co., Ltd. (Shanghai, China). All reagents utilized in the study were of analytical purity and prepared using distilled water.

### 2.2. Preparation of Kafirin

Sorghum seeds were ground into a fine powder. Soon thereafter, they were sieved through a 40-mesh graded test sieve and mixed with petroleum ether in a 1:5 (g/mL) ratio. This amalgamation was stirred at 500 rpm, employing the JJ-1 High Power Electric Stirrer from Changzhou Guohua Electric Appliances Co., Ltd. (Changzhou, China), at room temperature for a span of 6 h. After removing the petroleum ether by filtration and the drying of the pellets, the defatted sorghum powder was blended with a 70% ethanol solution containing 0.5% sodium metabisulfite and 0.35% sodium hydroxide. The mixture was allowed to stand at 70 °C for 1 h, and then centrifuged at 3000× *g* for 5 min in a HC-2064 high-speed centrifuge from Anhui Ustc Zonkia Scientific Instruments Co., Ltd. (Hefei, China). The supernatant was collected to reduce the ethanol concentration to 40%; then, the pH was adjusted to 5 and the mixture was allowed to stand at 0 °C to 4 °C for 6 h. The solution was then centrifuged at 3000× *g* for 5 min, and the resulting precipitate was washed three times with water. The precipitate was then freeze-dried under a vacuum to isolate the kafirin [16].

### 2.3. HHP Treatment of Kafirin

The kafirin was combined with distilled water at a ratio of 1:100 (g/mL), placed within a polyethylene bag, and subjected to vacuum packaging. The prepared sample was then immersed within a Sanshuihe SHPP-10 L HHP container from Shanxi Leadflow Technology Co., Ltd. (Jinan, China), with water serving as the pressurizing medium. The kafirin was treated at 100, 300, and 600 MPa for 10 min at a temperature of 25 °C and a pressurization rate of 200 MPa/min in the circulating water system, respectively. Then, the processed kafirin was freeze-dried and prepared for use [13].

### 2.4. Solubility

Protein solutions with ethanol concentrations of 10%, 20%, 30%, 40%, 50%, 60%, 70%, 80%, 90%, and 100% at 2 mg/mL were prepared, respectively. Using the Kjeldahl method, the protein content within the resulting supernatant was quantified, thus enabling the estimation of solubility. This solubility estimation was achieved by dividing the protein amount present in the supernatant by the total protein content [17]. Then, the concentration of ethanol that facilitated the maximum solubility was judiciously selected as the pivotal concentration for subsequent index calculations.

### 2.5. Sodium Dodecyl Sulfate–Polyacrylamide Gel Electrophoresis (SDS-PAGE)

SDS-PAGE was performed according to Laemmli’s method [18] using the DYY-7C electrophoresis apparatus (Liuyi Biological Technology Co., Ltd., Beijing, China). A 6 μL of 1 mg/mL of sample was added to 12% concentrate gel and 5% separator gel, and proteins were separated from the gels at a constant voltage (100 V for the concentrate gel and 150 V for the separator gel). At the end of the process, the gel was stained and decolorized, and then the gel was scanned and analyzed.

### 2.6. Ultraviolet Spectrum

UV–vis spectra of the samples (2 mg/mL in 70% ethanol) were collected from 250–300 nm at a rate of 60 nm/min using the Carry 60 UV–visible spectrophotometer (Agilent Technologies Co., Ltd., Shanghai, China). The second-order derivatives were calculated using the corresponding software Cary WinUV 5.0.0.999. The 70% ethanol solution was used as a blank [19].

### 2.7. Endogenous Fluorescence Spectrum

An RF-5301 fluorescence spectrophotometer, sourced from Shimadzu International Trading Co., Ltd. (Shanghai, China), facilitated the measurement of fluorescence in the kafirin solution with a concentration of 0.04 mg/mL in 70% ethanol. The excitation wavelength was set at 290 nm and the emission wavelength from 300 to 400 nm with 5 nm excitation and emission slits. A blank solution of 70% ethanol was utilized [20].

### 2.8. Particle-Size Distribution

Particle-size analysis was executed using the BT-9300ST laser particle-size-distribution device from Dandong Bettersize Instruments Co., Ltd. (Dandong, China). The process involved the use of distilled water as a dispersant, the addition of kafirin to achieve 10% opacity, and then ultrasonic treatment. The refractive index of the medium was set precisely at 1.33 [21].

### 2.9. Particle Size and Zeta Potential

Particle-size and zeta-potential analyses were completed using the Malvern Zetasizer Nano ZS90 instrument (Malvern, PA, USA). The testing protocol entailed generating a protein solution containing 2 mg/mL within 70% ethanol. The solution was passed through a 0.45 μm microporous filter membrane and equilibrated at 25 °C for 120 s before starting the assay [22].

### 2.10. Fourier Transform Infrared (FTIR) Spectrum

The samples were mixed with KBr in the ratio of 1:100 (*w*/*w*), ground and pressed, and then measured by a Tesor 27 FTIR spectrometer from Bruker Co. (Bremen, Germany), spanning the spectral range of 4000–400 cm^−1^. A total of 32 scans were executed at a resolution of 4 cm^−1^, and the acquired curves underwent fitting and analysis via the PeakFit 4.12 software [23].

### 2.11. Differential Scanning Calorimetry (DSC)

The measurement of differential scanning calorimetry (DSC) was carried out by the Mettler differential scanning calorimeter from Mettler-Toledo (Greifensee, Switzerland). For this test, approximately 3 mg of the sample was weighed and placed in a standard aluminum pot. The sample was heated to a temperature range spanning from 40 °C to 120 °C, with a controlled heating rate of 10 °C per minute. Additionally, a nitrogen flow rate of 50 mL/min was maintained within the reaction chamber. During this operation, an empty aluminum pot was used as a reference [24].

### 2.12. Surface and Total Free Sulfhydryl Group Content and Disulfide-Bond Content

Adhering to Yang’s methodology, a 0.1% solution of ethylenediaminetetraacetic acid (EDTA) containing 9 M urea was prepared. This solution was used as a solvent to prepare a 5 mg/mL protein solution; a 100 µL of dithionitrobenzoic acid (DTNB) solution at a concentration of 4 mg/mL was added to the 5 mL solution, and the mixture was stirred during 30 min at room temperature. This mixture was centrifuged for 10 min at 10,000× *g*, and in the obtained supernatant the absorbance was measured at 412 nm. For the determination of the total amount of free sulfhydryl groups, the extinction coefficient of 13,600 M^−1^ cm^−1^ was applied. Surface free sulfhydryl was assessed using a 0.1% ethylenediaminetetraacetic acid solution in the absence of 9 M urea [25].

Moreal’s technique was used to ascertain the disulfide-bond content. For the calculation of the thiol (SH) content, a protein solution of 5 mg/mL was prepared utilizing a Tris-Gly-8 M urea solution. An amount of 50 μL of the DTNB solution was added to 5 mL of this protein solution, and the mixture was allowed to react for a span of 1 h. After centrifugation at 10,000× *g* for 10 min, the supernatant was collected, and its absorbance was measured at 412 nm. The thiol equivalent (SHeq) was determined by weighing 15 mg of sample into 1.3 mL of the DTE solution, reacting at 60 °C for 2 h, and then adding 3 mL of glacial acetic acid to terminate the reaction. Postcentrifugation was performed at 10,000× *g* for 10 min and multiple washes with a glacial acetic acid solution; then, the precipitate was dissolved in 5 mL of Tris-Gly-8 M urea solution, and 50 µL of DTNB solution was added and the reaction was carried out for 1 h. After centrifugation at 10,000× *g* for 10 min, the supernatant was taken at 412 nm to determine its absorbance. The content of disulfide bonds was calculated using the equation SHeq = 2SS + SH [26].

### 2.13. Scanning Electron Microscope (SEM)

The specimens were carefully positioned onto the double-sided tape affixed to the carrier table. A gentle burst from the washout bulb was employed to eliminate any superfluous powder from the samples. Following this preparatory step, the samples were plated in gold before being examined using the Japanese Electronics Corporation JSM-7500 F scanning electron microscope [27].

### 2.14. Statistical Analysis

The experimental trials were replicated three times for each group, and the outcomes were presented as the mean value accompanied by the standard deviation. These results were graphically illustrated using Origin 8.0 software. Statistical analysis was carried out using Duncan’s method via the SPSS 18.0 software, employing a significance level set at *p* < 0.05.

## 3. Results and Discussion

### 3.1. Impact of the HHP Treatment on Solubility

**Figure 1:**
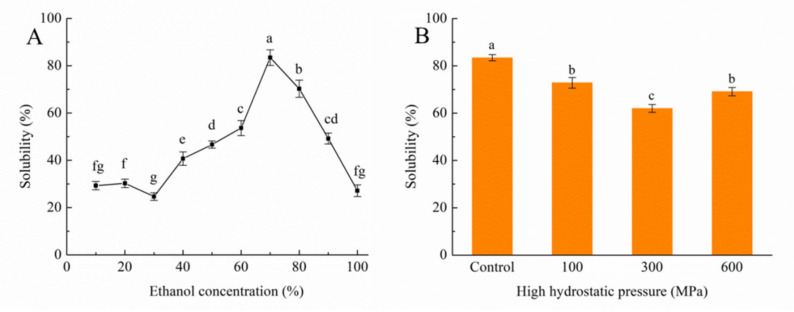
Solubility of kafirin in different ethanol concentrations (**A**); Solubility of kafirin in 70% ethanol before and after different HHP treatments (**B**); All experiments were repeated three times, and different letters indicate significant differences (*p* < 0.05).

Several attributes of proteins are influenced by their behavior in liquids, whether it be physical or chemical in nature. Of all these qualities, solubility is the most crucial. This is due to the fact that solubility has a significant impact on the physicochemical properties, processing techniques, sensory characteristics, shelf-life, and nutritional value of protein-rich foods. Furthermore, solubility plays an essential role in evaluating functional aspects, such as emulsification and foaming capabilities [28]. The solubility behavior of kafirin, as measured at varying concentrations of ethanol, is illustrated in Figure 1A. Notably, a decline in solubility is observed between ethanol concentrations of 10% and 30%, reaching its nadir at a 30% concentration. This trend then shifts as the solubility experiences a substantial surge within the 30% to 70% ethanol content range, culminating at 70%. An ensuing decrease in solubility is noted as the ethanol concentration reaches 100%. Therefore, the solvent chosen for subsequent index calculations is the 70% ethanol concentration. This behavior is attributed to the amphiphilic nature of kafirin, where the hydrophilic segment orients outward at the 70% ethanol concentration and, conversely, inward at the 30% ethanol concentration, inducing a decline in solubility [29].

The outcomes in Figure 1B illustrate a marked variance in kafirin solubility following distinct HHP treatments. Evidently, there is a downward trend in solubility with escalating pressure up to 300 MPa. However, a reversal occurred upon further elevation to 600 MPa, surpassing the solubility at 300 MPa, albeit still lower than that of the control group. This variation may be attributed to lower pressures inducing protein–protein interactions during the HHP treatment, leading to the formation of aggregates and solubility reduction [30]. Conversely, at higher pressures, these newly formed protein aggregates might experience partial disintegration [31].

### 3.2. Impact of the HHP Treatment on SDS-PAGE

**Figure 2:**
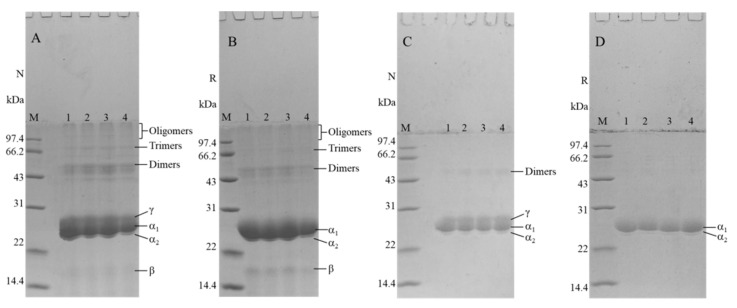
Nonreduced (**A**) and reduced (**B**) electropherograms of kafirin before and after treatment with different HHP treatments, and nonreduced (**C**) and reduced (**D**) electropherograms of supernatant dissolved in the 70% ethanol solution. M is the low-molecular-weight protein marker; lanes 1–4 are the control, 100 MPa, 300 MPa, and 600 MPa, respectively.

As evident in Figure 2A, the nonreduced profile displays discernible bands corresponding to γ-, α_1_-, α_2_-, and β-kafirin within the 14.4–31 kDa range, accompanied by bands signifying dimers, trimers, and oligomers, surpassing the 43 kDa threshold. A juxtaposition with the nonreduced profile showcases notable alterations in Figure 2B. The γ-kafirin band vanishes, while the bands associated with the dimers, trimers, and oligomers display a diminished intensity. Simultaneously, the hues of the β- and α_1_-kafirin bands intensify. This transformation is a consequence of introducing β-sulfanylethanol in the reduction profile, which effectively severs the disulfide bonds, leading to the disintegration of aggregates into monomeric constituents [32]. In Figure 2C, a comparison to Figure 2A reveals the disappearance of trimer, oligomer, and β-kafirin bands. Furthermore, the macromolecular aggregates fail to dissolve in a 70% ethanol solution. However, the reduction profiles portrayed in Figure 2D unveil the disappearance of dimer and γ-kafirin bands, accompanied by a deepening of α_1_-kafirin bands. These modifications result from the disruption of disulfide bonds facilitated by the introduction of β-sulfanylethanol. Notably, the bands representing subunits before and after distinct HHP treatments show no significant disparity. This observation underscores that HHP treatments do not induce changes in the subunit composition and that the apparent decrease in solubility is unrelated to potential alterations in the subunit specificity.

### 3.3. Impact of the HHP Treatment on the Ultraviolet Spectrum

**Figure 3:**
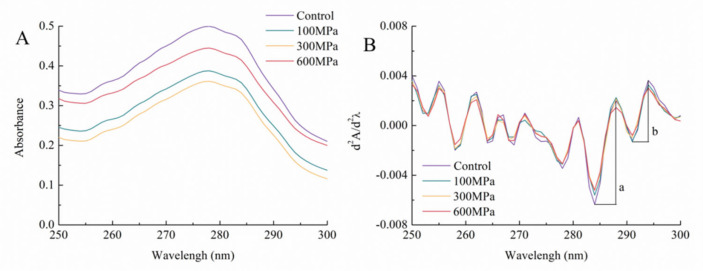
UV spectra (**A**) and second−order derivatives (**B**) of kafirin before and after different HHP treatments. The divergences between 284 nm and 288 nm are designated as “a” and the divergences between 291 nm and 294 nm are designated as “b”.

The UV-absorption spectrum primarily hinges on the presence of tyrosine and tryptophan residues within the side-chain structure. Additionally, residues of histidine, cysteine, and phenylalanine have a secondary affect [33]. As demonstrated in Figure 3A, the UV spectrum of kafirin, both before and after undergoing various HHP treatments, shows discernible absorption peaks at 278 nm within the 250–300 nm range. These peaks stem from the absorption of tryptophan and tyrosine residues, while no significant deviations appeared in the absorption-peak wavelengths among the different treatment groups.

Incorporating an array of aromatic amino acids, proteins provide a distinct overlap of a singular absorption band in the UV-absorption spectrum. Particularly, the second-order derivative spectrum possesses the capability to unveil alterations in the microenvironment surrounding tyrosine residues—an aspect not readily discernible in the conventional UV-absorption spectrum [34]. Figure 3B unveils the UV second-order derivative spectrum featuring troughs at 284 nm and 291 nm, coupled with peaks at 288 nm and 294 nm. The divergences between these peaks and troughs are designated as “a” and “b”. To capture changes in the microenvironment of tyrosine residues, the r-value is calculated based on the ratio of a/b. In specific terms, the control group, along with the 100 MPa, 300 MPa, and 600 MPa groups, exhibits r-values of 1.714, 1.720, 1.831, and 1.751, respectively. Additionally, as pressure escalates up to 300 MPa, the r-value demonstrates an upward trend, implying that the HHP treatment renders the tyrosine microenvironment within kafirin more polar and less hydrophobic. However, at the juncture of 600 MPa, the r-value begins to decline anew. This phenomenon is postulated to be linked to the hydrophobic burial effect of tyrosine residues under heightened pressure intensity, resulting in a hydrophobic arrangement. This observation aligns with the changes in the solubility of kafirin [35].

### 3.4. Impact of the HHP Treatment on the Endogenous Fluorescence Spectrum

**Figure 4:**
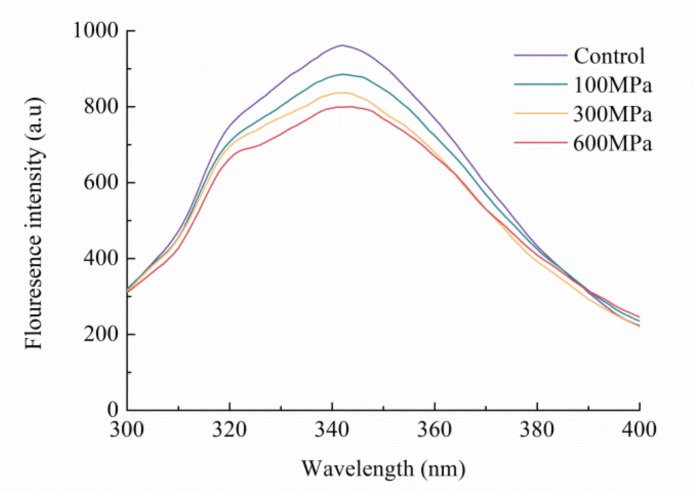
Endogenous fluorescence spectroscopy of kafirin before and after different HHP treatments.

Fluorescence spectroscopy provides a means to gauge alterations in the tertiary structure of proteins induced by environmental variations. Tryptophan residues’ natural fluorescence acts as a sensitive indication of the surrounding microenvironment [36]. As depicted in Figure 4, the control group displays its peak fluorescence intensity at 342 nm. As pressure increases, the maximum absorption wavelength experiences a slight redshift. Remarkably, at 600 MPa, the wavelength further redshifts from 342 nm to 344 nm, implying that tryptophan residues find themselves in a more polar milieu.

When interpreted alongside the findings derived from UV-spectrum analysis, it becomes evident that the imposition of the HHP treatment engenders an augmentation in the polarity of the microenvironment housing amino acids. This perturbation prompts their relocation to more hydrophilic domains, while concurrently attenuating the hydrophobic characteristics of kafirin. This interplay culminates in a discernible reduction in solubility.

### 3.5. Impact of the HHP Treatment on Particle-Size Distribution

**Figure 5:**
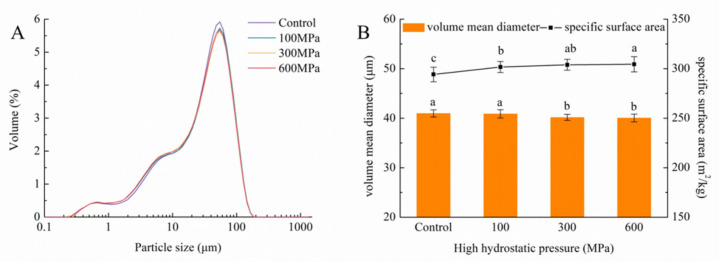
Size distribution in water (**A**); Volume mean diameter and specific surface area (**B**) of kafirin before and after different HHP treatments. All experiments were repeated three times, and different letters indicate significant differences (*p* < 0.05).

Laser particle sizing serves as a precise method for scrutinizing protein aggregation, offering insights into particle-size and distribution measurements [37]. Presented in Figure 5 are the outcomes of solid-particle-distribution measurements of kafirin within the aqueous phase. As illustrated in Figure 5A, discernible disparities in particle-size distribution pre- and post-HHP treatments are absent. Highlighted in Figure 5B, the volume average diameter of kafirin within an aqueous solution displays a gradual reduction in tandem with escalating pressure. In contrast, the specific surface area adopts a converse trend, progressively increasing. This phenomenon fosters heightened interaction between protein and water, leading to an augmented contact area and more uniformly dispersed protein–water molecule interaction [38]. These findings underscore that the HHP treatment amplifies the hydrophilic character of kafirin, thereby fostering a more uniform and stable system within an aqueous solution.

### 3.6. Impact of the HHP Treatment on Particle Size and Zeta Potential

**Figure 6:**
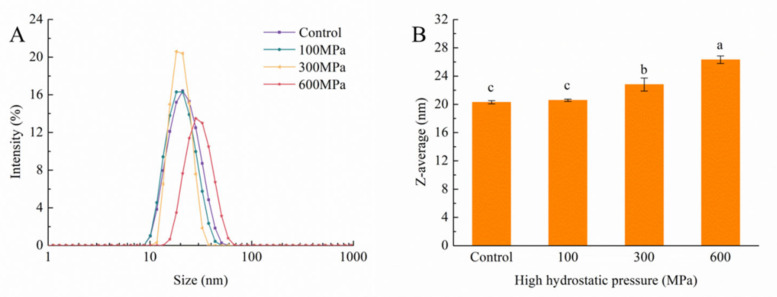
Particle-size distribution in 70% ethanol solution (**A**) and average particle size (**B**) of kafirin before and after different HHP treatments. All experiments were repeated three times, and different letters indicate significant differences (*p* < 0.05).

The stability of a solution finds a reflective expression through the assessment of the molecular particle size within it [39]. As evidenced in Figure 6A, the particle-size distribution before and after distinct HHP treatments presents a singular peak distribution ranging from 10 to 100 nm in the 70% ethanol solution. A comparison with the control group reveals a discernibly narrower peak width at 300 MPa, demonstrating an increased prevalence of particles of similar size and an enhanced overall solution homogeneity. Extremely, at 600 MPa, the particle-size-distribution peak shows a pronounced inclination towards larger particle sizes, coinciding with a notable elevation in the average particle-size value. These findings imply that the HHP treatment influences noncovalent interactions among protein molecules, prompting the reconfiguration of inter- or intramolecular valence bonds, ultimately fostering kafirin aggregation [40]. Importantly, this pattern diverges from the particle-size distribution noticed within the aqueous phase. In this context, the HHP treatment prompts the expansion of protein particle size and aggregation in the 70% ethanol solution while, conversely, particle-size values decrease in the aqueous solution. This discrepancy highlights that the HHP treatment leads to a modulation of kafirin’s hydrophobicity, favoring an augmented hydrophilic character.

**Figure 7:**
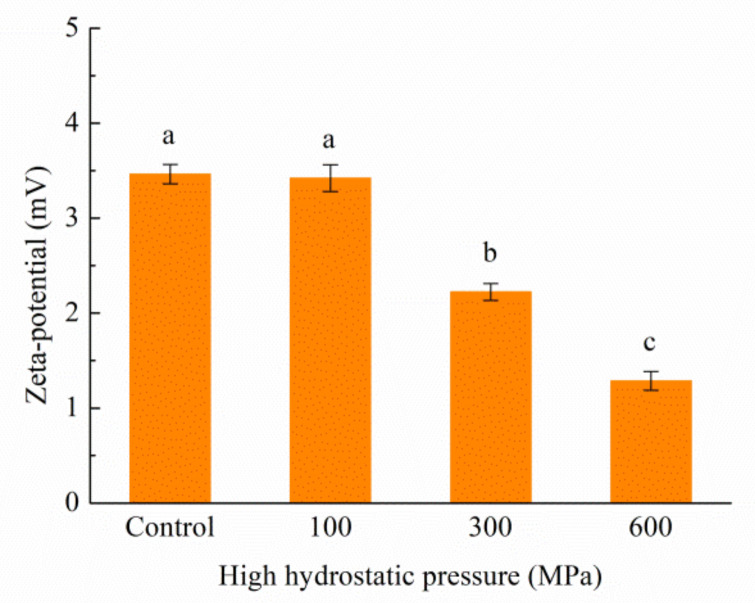
Zeta potential of kafirin before and after different HHP treatments. All experiments were repeated three times, and different letters indicate significant differences (*p* < 0.05).

The concept of zeta potential elucidates the magnitude of charges inherent within a solution, exerting a significant impact on its stability. The effective charge within a protein solution is a function of the ionization of diverse amino acid residues, pH, and ionic strength [41]. As depicted in Figure 7, the measured zeta-potential values of the solutions exhibit a positive charge, indicative of a higher count of positively charged amino acids in contrast to negatively charged ones on the kafirin surface. Indeed, the zeta-potential values for the 300 MPa and 600 MPa treatments significantly diverge from those of the control group. This discovery suggests that the ethanol solution curtails electrostatic interactions among proteins, thereby fostering particle aggregation and a resultant increase in particle-size values.

### 3.7. Impact of the HHP Treatment on the FTIR Spectrum

FTIR spectroscopy serves as a revealing tool for unraveling the unfolding and molecular-level structural reconfiguration of proteins. The comprehensive analysis of amide I bands, detailed in Table 1, is instrumental in dissecting the secondary structure of proteins. These bands are particularly valuable, encompassing intermolecular β-sheets (1610–1625 cm^−1^ and 1685–1695 cm^−1^), intramolecular β-sheets (1625–1640 cm^−1^ and 1670–1684 cm^−1^), random coils (1640–1648 cm^−1^), α-helices (1648–1658 cm^−1^), and β-turns (1660–1668 cm^−1^) [42].

The secondary structure of kafirin is notably dominated by β-sheets, with intermolecular β-sheet content playing a prominent role. Prior to and subsequent to various HHP treatments, there is no significant alteration in the content of random coils. Moreover, the constituent content of secondary-structure components closely aligns with that of the control group at lower pressures (100 MPa) without displaying significant divergence. However, an intriguing transformation occurs within the secondary structure as pressure escalates to 300 MPa and 600 MPa. In particular, the transition transpires from an intramolecular to an intermolecular configuration, with α-helices and β-turns yielding their place to β-sheets. This shift toward intermolecular β-sheets is indicative of heightened protein aggregation [43]. Consequently, these results underscore that the HHP treatment is conducive to enhancing the organization and aggregation of the kafirin structure.

### 3.8. Impact of the HHP Treatment on DSC

DSC, a thermodynamic technique, orchestrates the transition of proteins from their inherent conformation to a denatured state during the heating process. This transformation is accompanied by the disruption of inter- and intramolecular valence bonds. Central to this process is the denaturation temperature, a major indicator used to gauge the thermal stability of proteins. The higher denaturation-peak temperatures correspond to heightened thermodynamic stability within the protein structure [44].

**Figure 8:**
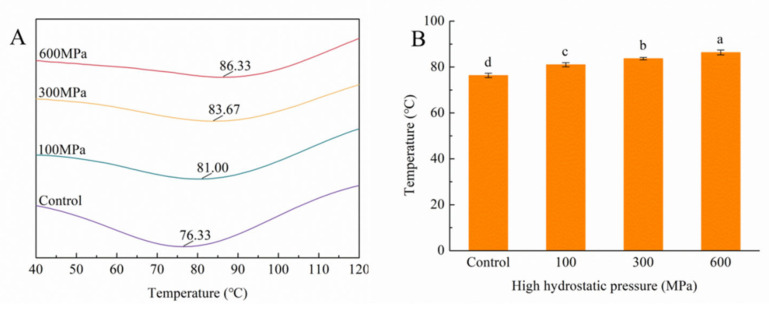
Peak denaturation temperature curve (**A**) and values (**B**) of kafirin before and after different HHP treatments. All experiments were repeated three times, and different letters indicate significant differences (*p* < 0.05).

Figure 8 furnishes the peak denaturation temperatures to HHP treatments. Evidently, as pressure levels increase, the denaturation temperature of kafirin registers a noteworthy elevation, surging from 76.33 °C to 86.33 °C. This substantial increment explains an enhancement in the thermal stability of the protein. It is established that proteins endowed with a more compact structure tend to exhibit higher denaturation temperatures [30]. These discernible results, when amalgamated with the secondary-structure measurements, reflect the transformative influence of the HHP treatment on the kafirin conformation. This alteration paves the way for improved order and stability within the kafirin structure.

### 3.9. Impact of HHP Treatments on Surface Free Sulfhydryl Groups, Total Free Sulfhydryl Groups, and Disulfide-Bond Content

**Figure 9:**
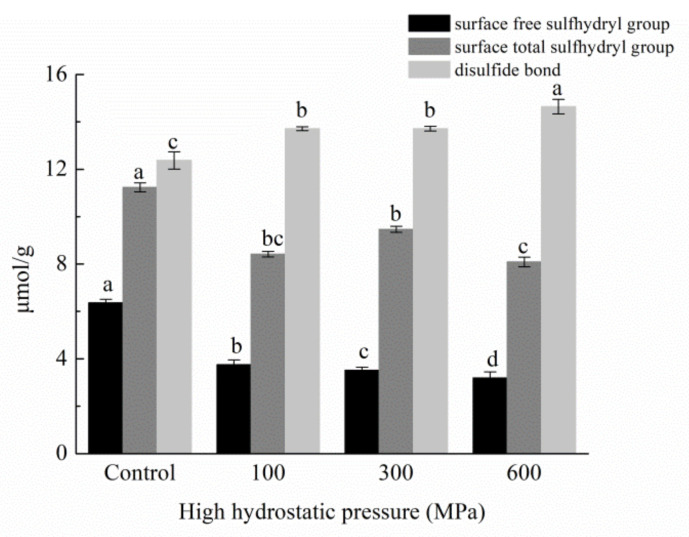
Free and total sulfhydryl group and disulfide-bond content of kafirin before and after different HHP treatments. All experiments were repeated three times, and different letters indicate significant differences (*p* < 0.05).

The integrity of a protein’s tertiary structure is upheld through sulfhydryl groups and disulfide linkages, and modifications in these structures are intrinsically tied to the extent of protein denaturation, thus wielding a substantial impact on its functional attributes [45]. In Figure 9, the variations in surface free sulfhydryl groups, total free sulfhydryl groups, and disulfide bonds are elucidated both prior and subsequent to distinct HHP treatments. Significantly, the escalation of pressure engenders a pronounced reduction in the contents of free sulfhydryl groups and total sulfhydryl groups. In stark contrast, the content of disulfide bonds registers a significant increase. These observations converge to suggest that, during the HHP treatment, intermolecular and intramolecular protein chains form disulfide bonds via sulfhydryl and disulfide-bond exchange reactions. When paired with the decrease in α-helix content and the concomitant rise of β-sheet components within the secondary structures, a compelling narrative emerges. This narrative implies a constrained degree of freedom for individual protein chains and a concomitant enhancement in protein molecule stability [46].

### 3.10. Impact of the HHP Treatment on SEMs

**Figure 10:**
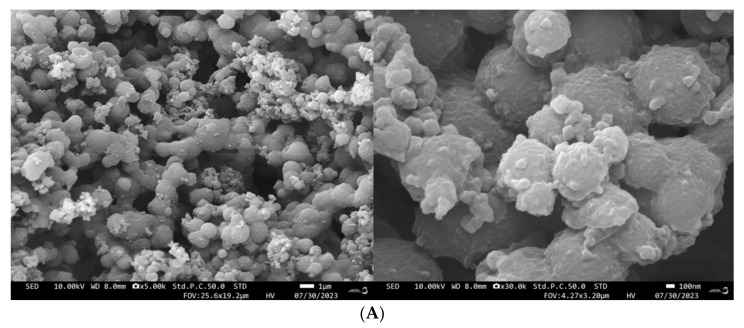

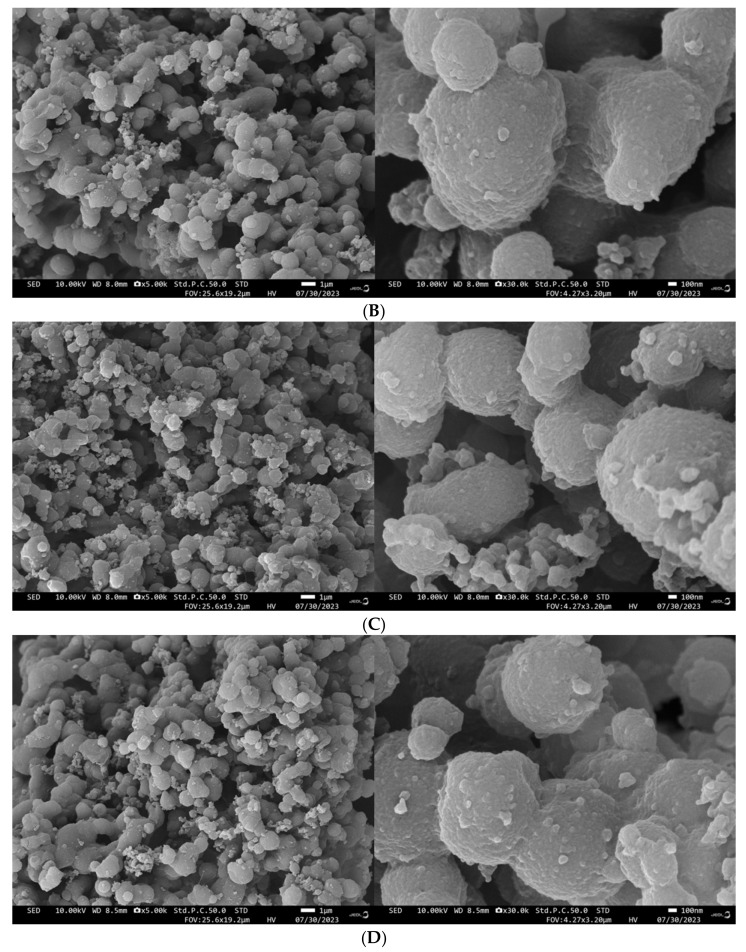
Scanning electron microscopy left (5 k) and right (30 k) of kafirin before and after different HHP treatments ((**A**): Control; (**B**): 100 MPa; (**C**): 300 MPa; (**D**): 600 MPa).

Transitioning to the SEM analysis, a visual comparison between the control group (Figure 10A) and kafirin subjected to the HHP treatment (100, 300, and 600 MPa) is unveiled in Figure 10B–D. Each figure encompasses scanning electron micrographs at two different magnifications (×5000 and ×30,000). The images reveal a topography characterized by the distribution of small particles atop larger particles, along with the aggregation or adherence of smaller particles between larger ones, thus assuming an uneven spherical morphology. Upon magnification to ×30,000, it becomes evident that the HHP treatment engenders a more uniform and compact distribution of protein particles, coupled with heightened aggregation between the particles.

## 4. Conclusions

Various aspects of kafirin were subjected to scrutiny under distinct HHP treatments, encompassing solubility, subunit composition, particle-size distribution, thermal stability, secondary structure, disulfide-bond content, and microstructure. The cumulative findings unveiled a compelling narrative. Notably, the HHP treatment engendered a reduction in kafirin’s solubility within a 70% ethanol solution, while abstaining from revealing any conspicuous subunit aggregation. Intriguingly, these treatments imparted a heightened polarity to the microenvironments enveloping tyrosine, tryptophan, and other chromophores. This shift resulted in an augmented average particle size for protein molecules within the 70% ethanol solution, coupled with a decrease in the potential value. In concert, scanning electron microscopy analyses illustrated the propensity of protein molecules to aggregate in this context. A counterpoint emerged when assessing solid particles within the aqueous phase: an attenuation in the average particle-size value, juxtaposed with an increase in specific surface area. These findings collectively hinted at an amplification in kafirin’s hydrophilicity, concomitant with a weakening of its hydrophobic characteristics. Further insights emerged when evaluating the denaturation temperature of kafirin. The HHP treatment effectively elevated this temperature, indicative of a more ordered and stable structure. This transformation was mirrored by the amplified content of β-sheet and disulfide bonds. These cumulative revelations paint a vivid picture of kafirin undergoing a transition toward heightened organization and stability as a result of the HHP treatment.

## Figures and Tables

**Table 1:** Secondary structure of kafirin before and after different HHP treatments.

High Hydrostatic Pressure	Comparative Content/%				
	Intermolecular β-Sheet	Intramolecular β-Sheet	Random Coil	α-Helix	β-Turn
Control	19.97 ± 0.17 ^b^	41.06 ± 0.05 ^a^	12.66 ± 0.01 ^a^	13.52 ± 0.07 ^ab^	12.79 ± 0.05 ^ab^
100 MPa	19.95 ± 0.33 ^b^	40.76 ± 0.13 ^ab^	12.51 ± 0.11 ^a^	13.78 ± 0.13 ^a^	13.00 ± 0.06 ^a^
300 MPa	20.55 ± 0.36 ^ab^	40.58 ± 0.09 ^b^	12.45 ± 0.09 ^a^	13.55 ± 0.16 ^ab^	12.87 ± 0.16 ^ab^
600 MPa	21.06 ± 0.91 ^a^	40.59 ± 0.36 ^b^	12.29 ± 0.37 ^a^	13.39 ± 0.29 ^b^	12.67 ± 0.27 ^b^

Notes: All experiments were repeated three times, and different letters indicate significant differences (*p* < 0.05).

## Data Availability

The data presented in this study are available on request from the corresponding author.
